# C-Reactive Protein Impairs Dendritic Cell Development, Maturation, and Function: Implications for Peripheral Tolerance

**DOI:** 10.3389/fimmu.2018.00372

**Published:** 2018-03-05

**Authors:** Rachel V. Jimenez, Tyler T. Wright, Nicholas R. Jones, Jianming Wu, Andrew W. Gibson, Alexander J. Szalai

**Affiliations:** ^1^Department of Medicine, Division of Clinical Immunology & Rheumatology, University of Alabama at Birmingham, Birmingham, AL, United States; ^2^Division of Drug Development, Southern Research, Birmingham, AL, United States; ^3^Department of Veterinary and Biomedical Sciences, University of Minnesota, Minneapolis, MN, United States

**Keywords:** acute phase response, aging, autoimmunity, inflammaging, inflammation, transgenic

## Abstract

C-reactive protein (CRP) is the prototypical acute phase reactant, increasing in blood concentration rapidly and several-fold in response to inflammation. Recent evidence indicates that CRP has an important physiological role even at low, baseline levels, or in the absence of overt inflammation. For example, we have shown that human CRP inhibits the progression of experimental autoimmune encephalomyelitis (EAE) in CRP transgenic mice by shifting CD4^+^ T cells away from the T_H_1 and toward the T_H_2 subset. Notably, this action required the inhibitory Fcγ receptor IIB (FcγRIIB), but did not require high levels of human CRP. Herein, we sought to determine if CRP’s influence in EAE might be explained by CRP acting on dendritic cells (DC; antigen presenting cells known to express FcγRIIB). We found that CRP (50 µg/ml) reduced the yield of CD11c^+^ bone marrow-derived DCs (BMDCs) and CRP (≥5 μg/ml) prevented their full expression of major histocompatibility complex class II and the co-stimulatory molecules CD86 and CD40. CRP also decreased the ability of BMDCs to stimulate antigen-driven proliferation of T cells *in vitro*. Importantly, if the BMDCs were genetically deficient in mouse FcγRIIB then (i) the ability of CRP to alter BMDC surface phenotype and impair T cell proliferation was ablated and (ii) CD11c-driven expression of a human *FCGR2B* transgene rescued the CRP effect. Lastly, the protective influence of CRP in EAE was fully restored in mice with CD11c-driven human FcγRIIB expression. These findings add to the growing evidence that CRP has important biological effects even in the absence of an acute phase response, i.e., CRP acts as a tonic suppressor of the adaptive immune system. The ability of CRP to suppress development, maturation, and function of DCs implicates CRP in the maintenance of peripheral T cell tolerance.

## Introduction

Inflammation is a normal local response to tissue injury and infection. If the insult is sufficiently strong there will follow a systemic response, termed the acute phase response (APR), during which leukocytes release inflammatory mediators (primarily IL-6, IL-1, and/or TNFα) into the circulation that sequentially propel a diversity of effects. During the APR, the liver increases the synthesis of a number of pattern recognition proteins. Among these C-reactive protein (CRP) is the prototype; it is maintained at low levels in normal sera (1–5 µg/ml) (1), but can reach upwards of ~500 μg/ml during inflammation (2). CRP’s ability to activate complement, opsonize microbes, bind to phosphatidylserine on apoptotic cells, and bind Fc receptors is well known (2–4) and these biological actions have been studied extensively in the context of CRP’s upregulation during inflammation. Increasing evidence indicates that CRP also exerts important biological influences even when its levels remain low as in healthy individuals and when it is only slightly raised as in aging individuals (4).

Previously, we have shown that human CRP transgenic mice (CRPtg) are resistant to experimental autoimmune encephalomyelitis (EAE), a disease comparable to human multiple sclerosis (MS) i.e., they have delayed onset of disease and milder clinical symptoms compared to wild type (WT) mice. Notably, despite the ability of CRPtg to mount a robust human CRP acute phase response, this protection does not require high levels of human CRP. We initially attributed CRP’s protective action in EAE to inhibition of encephalitogenic T cells, since *in vitro* CRP reduced T cell proliferation and shifted their cytokine production toward a less noxious T_H_2 profile (5). Our subsequent studies demonstrated that FcγRIIB^−/−^ mice, which lack expression of this inhibitory receptor, were refractory to CRP’s protective action (6), but we did not identify the FcγRIIB-expressing cell(s) that CRP relied upon. Herein, we demonstrate that CRP impairs the development of bone marrow (BM) cells into CD11c^+^ dendritic cells (DCs), professional antigen presenting cells that express ample FcγRIIB (7), are paramount for robust T cell responses (8), and are known to contribute to EAE/MS (9, 10, 11).

At doses as low as 5 µg/ml, CRP significantly prohibited bone marrow-derived DCs (BMDC) activation/maturation in response to stimulation with lipopolysaccharide (LPS), and impaired the ability of BMDCs to promote antigen-specific T cell proliferation. These suppressive actions of CRP were not evident using FcγRIIB^−/−^ BMDCs, but BMDCs from FcγRIIB^−/−^ mice genetically reconstituted to express a CD11c-driven human FcγRIIB transgene (_cd11c_FcγRIIB^hu^) were responsive to CRP, i.e., CRP prohibited their activation/maturation in response to LPS and suppressed their ability to promote T cell proliferation. As we previously reported, CRPtg were more resistant to EAE compared to WT, whereas CRPtg lacking expression of endogenous FcγRIIB (FcγRIIB^−/−^/CRPtg), were not. For the latter, however, expression of the CD11c-specific human FcγRIIB transgene fully reconstituted human CRP-mediated protection from EAE.

Based on these new findings, we propose that CRP acts as an endogenous down-regulator of DC development and activation/maturation, thereby acting as a brake on T cell mediated immunity and shifting the balance toward tolerance. Given that many of the effects of CRP on DCs were observed using ≤10 μg/ml, it is likely that even modest elevation of blood CRP—like that associated with aging (12)—is sufficient to significantly affect T cell tolerance.

## Materials and Methods

### Mice

Our human CRPtg have been fully described elsewhere (13, 14). In brief, CRPtg (C57BL/6 background) carry a 31-kb human DNA fragment encoding the *CRP* gene and all the *cis*-acting elements required for tissue specificity and acute phase inducibility, while the *trans*-acting factors required for its human-like pattern of regulation are conserved from mouse to man (13, 14). Consequently, unlike WT, CRPtg exhibit a robust human CRP acute phase response during inflammation. FcγRIIB deficient mice (FcγRIIB^−/−^; B6.129S4-*Fcgr2b^tm1TtK^* N12) (15) were purchased from Taconic Farms (Germantown, NY). 2D2 mice [C57BL/6-Tg(Tcra 2D2, Tcrb 2D2) 1Kuch/J] (16) are transgenic for a T cell receptor (TCR) that recognizes residues 35–55 of myelin oligodendrocyte glycoprotein (MOG_35–55_) and were purchased from Jackson Laboratories (Bar Harbor, ME, USA; JAX 006912). OT-II mice [B6.Cg-Tg(Tcra Tcrb)425Cbn/J] (17) are transgenic for a TCR that recognizes residues 323–339 of ovalbumin (OVA_323–339_) and were purchased from Jackson Laboratories (Bar Harbor, ME, USA; JAX 004194). FcγRIIB^−/−^ mice expressing a human *FCGR2B* transgene driven by a mouse CD11c minimal promoter (_cd11c_FcγRIIB^hu^) were generated herein and are fully described in the Section “Results.” To date, no embryonic lethality or unusual phenotype has been observed for _cd11c_FcγRIIB^hu^. C57BL/6 mice (WT) were obtained from the Jackson Laboratories (Bar Harbor, ME, USA; JAX 000664). All mice were housed in the same vivarium at constant humidity (60 ± 5%) and temperature (24 ± 1°C) with a 12-h light cycle (6:00 a.m. to 6:00 p.m.), and maintained *ad libitum* on sterile water and regular chow (Harlan Teklad). Mice were 8–12 weeks old when used and both sexes were combined unless specifically noted. All animal use protocols were approved by the Institutional Animal Care and Use Committees at the University of Alabama at Birmingham and were consistent with the *Guide for the Care and Use of Laboratory Animals; Eighth Edition* (NIH Academies Press, 2011).

### BMDC Cultures

Bone marrow progenitors were grown under conditions known to drive DC generation and expansion (18, 19). Briefly, BM was harvested from femurs, the red blood cells lysed (Hybri-Max Red Blood Cell Lysing Buffer; Sigma, Salem, MA, USA), and the marrow passed through a 70 µM cell strainer and brought to single-cell suspension in RPMI 1640 (Gibco, Grand Island, NY) containing 5% fetal bovine serum (Gibco), 1% Penicillin/Streptomycin (Gibco), 2 mM GlutaMAX™ (Invitrogen), non-essential amino acids (Gibco), 55 µM β-mercaptoethanol (Gibco), and 20 ng/ml granulocyte macrophage-colony stimulating factor (Shenandoah Biotechnology, Warwick, PA, USA). BM progenitors were then added to 12-well tissue culture plates (1 × 10^6^ cells in 1 ml per well) that were incubated at 37°C, 5% CO_2_ for 7 days. The culture medium was replaced on days 3 and 5. On day 5, cells were exposed to 50 µg/ml of highly purified human CRP (endotoxin and azide-free CRP from US Biological; Salem, MA, USA), purified chicken OVA_323–339_ peptide (MISC-011; CPC Scientific, San Jose, CA, USA), or purified MOG_35–55_ peptide (12668-01; Biosynthesis Inc., Lewisville, TX, USA). OVA_323–339_ and MOG_35–55_ loaded BMDCs were subsequently used in BMDC:T cell co-cultures with OT-II and 2D2 T cells, respectively, as described below. To trigger BMDC maturation in some experiments LPS from *Escherichia coli*, serotype 055:B5 (Sigma Aldrich) was added (1 µg/ml) on day 6. Alternatively, culture medium was supplemented with 100 ng/ml interleukin-4 (IL-4; Shenandoah Biotechnology, Warwick, PA, USA). IL-10 and IL-12p70 production was assessed by ELISA (88-7105-22 and 88-7121-22; Invitrogen, Eugene, OR, USA) according to the manufacturer protocol. Flow cytometry was performed on a BD LSR-II cytometer (described below) and, after excluding dead cells and aggregated cells, BMDCs were identified as CD11b^+^ CD11c^+^ cells. For detailed analysis of cell death, cells were stained with Annexin V and 7-AAD and were defined as early apoptotic (Annexin V^+^ 7-AAD^−^), late apoptotic (Annexin V^+^ 7-AAD^+^), necrotic (Annexin V^−^ 7-AAD^+^), or live (Annexin V^−^ 7-AAD^−^).

### T Cells and BMDC:T Cell Cocultures

From OT-II and 2D2 mice, the spleens and lymph nodes (axillary, brachial, inguinal) were harvested and mechanically homogenized, the red blood cells lysed, and the homogenate passed through a 70 µM cell strainer, and brought to single-cell suspension in media at 1 × 10^8^ cells/ml. CD4^+^ T cells were enriched by negative selection according to the manufacturer’s guidelines using a kit from StemCell Technologies (Vancouver, BC, Canada). Enriched CD4^+^ T cells were then stained for 20 min with 1 µM CellTrace™ carboxyfluorescein succinimidyl ester (CFSE; Invitrogen, Eugene, OR, USA). BMDCs, cultured as described above, were treated with MOG_35–55_ or OVA_323–339_ peptide on day 6. On day 7, the peptide-loaded BMDCs were mixed with the freshly isolated and CFSE-stained CD4^+^ T cells (1:5 ratio in triplicate), placed into 96-well round bottom plates, and incubated for 3 days before analysis of CD4^+^ T cell proliferation (dilution of CFSE). BMDC:T cell co-cultures exposed to plate-bound anti-CD3ε and soluble anti-CD28 antibodies (both from Biolegend, San Diego, CA, USA) served as positive controls.

### Antibodies and Flow Cytometry

Cells were washed with PBS, spun down at 300 × *g* for 5 min at 4°C, stained with the viability dye eFluor 780 (eBioscience, San Diego, CA, USA) for 30 min at room temperature, fixed in Fixation Buffer (Biolegend, San Diego, CA, USA) for 10 min at room temperature, blocked with anti-mouse CD16/32 (clone 93; eBioscience) at 4°C for 15 min, and stained with specific antibodies at 4°C for 30 min. For BMDCs, we used anti-mouse CD11c (clone N418), MHC class II IA/IE (clone M5/114.15.2), CD40 (clone HM40-3), CD80 (clone 16–10 A1), CD86 (clone GL-1) (all from Biolegend), and FcγRIIB (clone AT 130-5, Bio Rad, Hercules, CA, USA), and anti-human FcγRIIB (clone AT 10, AbD Serotec, Raleigh, NC, USA). For T cells we used anti-mouse CD4 (clone RM4-5) (Biolegend). Stained and labeled cells were run on a BD LSR-II cytometer and the acquired data analyzed using BD FACSDiva version 6.1.3 and FlowJo version 10.3. For all gating analyses, debris was gated out using a FSC by SSC dot plot, followed by selection of single cells using a SSC-A by SSC-H dot plot, and live cells were selected based on the viability dye eFluor 780 dot plot. For assessment of T cell proliferation (CFSE dilution), the bounds for the CD4^+^ CFSE^+^ “parents” gate was determined using unstimulated T cells and the bounds for the “progeny peaks” were based on anti-CD3ε/anti-CD28 stimulated T cells (see Figure 3A). As T cells divide, the progeny:parent ratio increases.

### Experimental Autoimmune Encephalomyelitis

Experimental autoimmune encephalomyelitis was induced as we described previously (5, 6, 20). Briefly, 10–12-week-old mice were immunized subcutaneously with 150 µg MOG_35–55_ emulsified in Freund’s complete adjuvant plus 400 µg heat-killed *Mycobacterium tuberculosis* (Difco, Detroit, MI, USA). On days 0 and 2, mice received an intraperitoneal injection of 200 ng pertussis toxin (List Biological Laboratories, Campbell, CA). For 30 days thereafter the development of EAE was monitored daily. EAE symptoms were scored on a clinical scale ranging from 0 to 6 as follows: 0, asymptomatic; 1, loss of tail tone; 2, flaccid tail; 3, incomplete paralysis of one or two hind limbs; 4, complete hind limb paralysis; 5, moribund (at which case the mouse was humanely euthanized); 6, dead. For mice that developed EAE, the day of onset was defined as the first of two consecutive days, wherein the clinical score was ≥2.

### Statistical Analysis

Raw data were pooled and are expressed graphically as the mean ± SEM or SD, as noted. Group comparisons were done using one-way analysis of variance (*ANOVA*) followed by *post hoc* Bonferroni’s and Tukey’s multiple comparison tests, or using linear trends tests. Differences were considered significant when *p* was <0.05. For EAE, the maximum clinical score achieved by each animal during the 30-day observation period was used to calculate the average maximum clinical score (a measure of severity). To study the time-course of disease, average clinical scores were calculated and plotted daily for each group of mice, and cumulative disease index (CDI) was calculated by area under the curve analysis. Statistical analyses were done using GraphPad Prism version 7.00.

## Results

### CRP Suppresses Generation and Maturation of BMDCs

**Figure 1:**
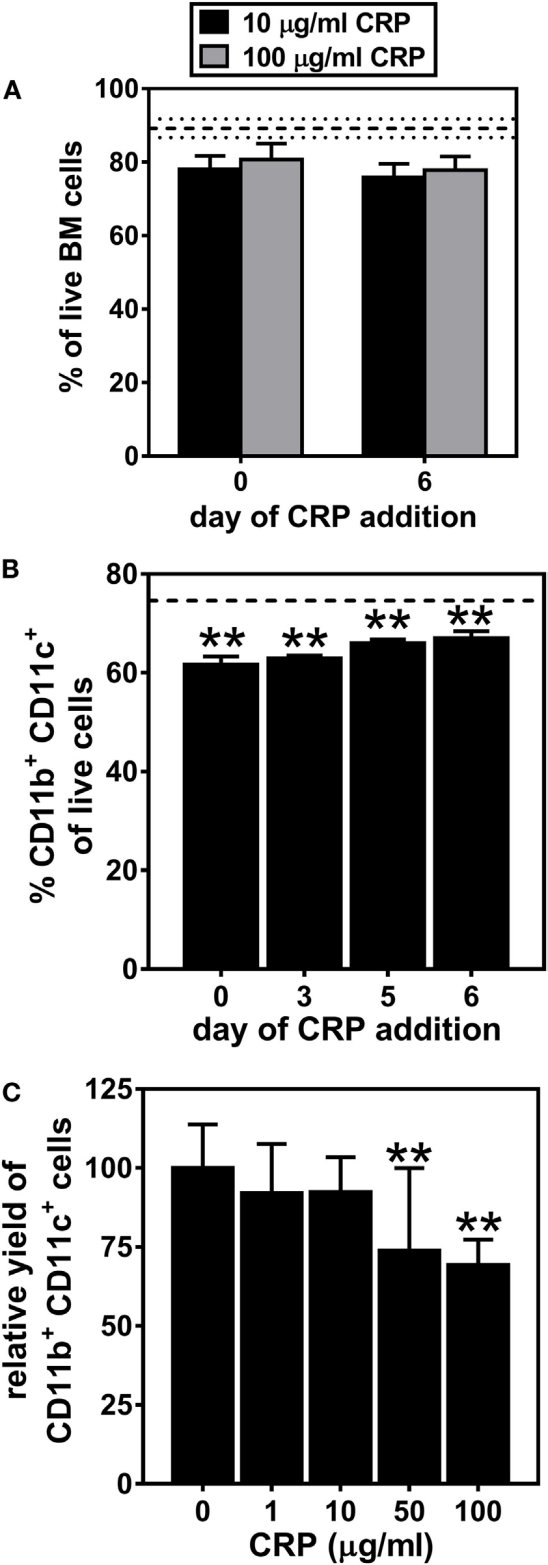
C-reactive protein (CRP) impedes the generation of CD11b^+^ CD11c^+^ bone marrow-derived dendritic cells (BMDCs) in a temporal and dose-dependent fashion. **(A)** CRP addition to bone marrow cultures on day 0 or on day 6 had no significant effect on cell viability. The horizontal dashed lines indicate cell viability of 89.2% ± 2.52 (mean ± SD) without CRP. **(B)** The proportion of live cells that were CD11b^+^ CD11c^+^ BMDCs was significantly reduced by addition of CRP (50 µg/ml) on the indicated day of culture. The horizontal dashed line indicates the average proportion of BMDCs generated in the absence of CRP (74.6% ± 0.57 SD). **(C)** The relative yield of CD11c^+^ BMDCs was reduced in a dose-dependent fashion by CRP (1–100 µg/ml) added on day 0 of culture. The symbols indicate the results of one-way analyses of variance with Tukey’s multiple comparisons tests compared to cultures not treated with CRP, *p* < 0.005 (**) (*n* = 3–9 per group).

We first examined the influence of human CRP on the generation of DCs from BM progenitors. On day 7 of culture, nearly 90% of all cells were viable (dashed horizontal lines in Figure 1A) and the cultures routinely achieved a yield of nearly 75% BMDCs (dashed horizontal line in Figure 1B). Whether CRP at 10 or 100 µg/ml was added on day 0 or 6 of culture it had no significant effect on cell viability (Figure 1A). CRP treatment also had no effect on the proportion of early apoptotic, late apoptotic, and necrotic BM cells (data not shown). However, CRP treatment did significantly decrease (by 10–15%) the proportion of CD11b^+^ CD11c^+^ BMDCs that developed (Figure 1B). Notably, when CRP was added at the initiation of culture, the inhibitory effect on the final yield of BMDCs was strongest (Figure 1B) and was dose-dependent (Figure 1C). These results show that while CRP has no significant influence on the viability of cultured BM progenitors, it does significantly impede the generation of CD11b^+^ CD11c^+^ BMDCs in both a temporal and dose-dependent manner.

**Figure 2:**
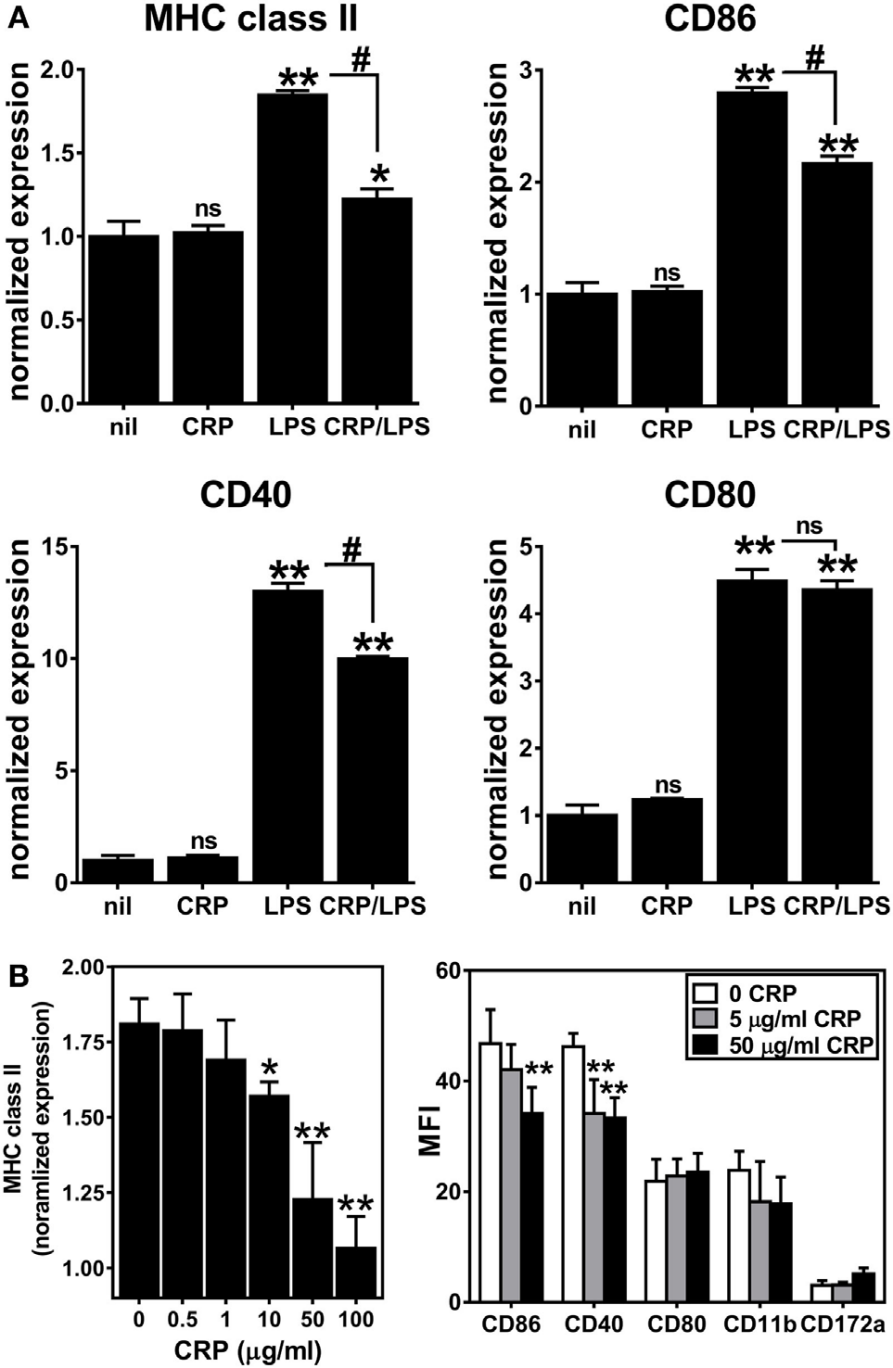
C-reactive protein (CRP) suppresses expression of MHC class II, CD86, and CD40 on lipopolysaccharide (LPS)-matured bone marrow-derived dendritic cells (BMDCs) in a dose-dependent manner. **(A)** Surface expression of MHC class II, CD86, CD40, and CD80 on immature CD11b^+^ CD11c^+^ BMDCs left untreated (nil) or treated with CRP (50 µg/ml on day 5), and on BMDCs matured with LPS (1 µg/ml on day 6) or treated with CRP (50 µg/ml on day 5) and LPS (1 µg/ml on day 6) (CRP/LPS). Expression of each marker (MFI of flow cytometry) is normalized to expression on untreated immature BMDCs (nil). The symbols above each bar indicate not significant (ns), *p* < 0.05 (*), or *p* < 0.005 (**) compared to “nil.” The symbols above each bracket indicate ns or *p* < 0.005 (#) for the LPS versus CRP/LPS groups. One-way analyses of variance (ANOVAs) with Tukey’s multiple comparisons tests. **(B)** CRP dose-dependent suppression of expression of MHC class II (left) and CD86 and CD40 (right) by LPS-treated BMDCs. MHC class II expression is normalized as in **(A)**. The symbols indicate the results of one-way ANOVAs with Tukey’s multiple comparisons tests, *p* < 0.05 (*) and *p* < 0.005 (**) compared to no CRP (*n* = 2–6 per group).

Next, we assessed the influence of CRP on activation/maturation of BMDCs. Treating immature BMDCs with CRP (50 µg/ml) had no effect on their surface expression of MHC class II, CD86, CD40, and CD80 (Figure 2A), whereas treatment of immature BMDCs with LPS (1 µg/ml) significantly upregulated these markers (Figure 2A), indicative of BMDC maturation. While CRP did not trigger BMDC maturation, CRP did significantly inhibit the LPS-triggered increase in surface expression of MHC class II and the co-stimulatory markers, CD86 and CD40 (Figure 2A). Also, the suppressive effect of CRP on LPS-triggered BMDC maturation was dose-dependent, as evidenced by a stepwise reduction of MHC class II, CD86, and CD40 (Figure 2B). This suppressive effect was specific as CRP had no effect on the expression of CD80, CD11b, or CD172a (Figure 2B). Finally, BMDCs treated with LPS (1 µg/ml) robustly produced both the T cell suppressive cytokine IL-10 and the T cell stimulatory cytokine IL-12p70 (225.7 ± 8.8 and 1245.8 ± 191.0 ng/ml, respectively), but the production of both cytokines was significantly suppressed by CRP (no detectable IL-10 and 773.2 ± 13.2 ng/ml IL-12p70; *p* < 0.05, *t*-tests). These data demonstrate that CRP dose-dependently prohibits LPS-triggered activation/maturation of BMDCs and limits their production of IL-10 and IL-12p70, cytokines with pleiotropic effects in immunoregulation.

### CRP Inhibits BMDC-Mediated Stimulation of Antigen-Specific T Cell Proliferation

**Figure 3:**
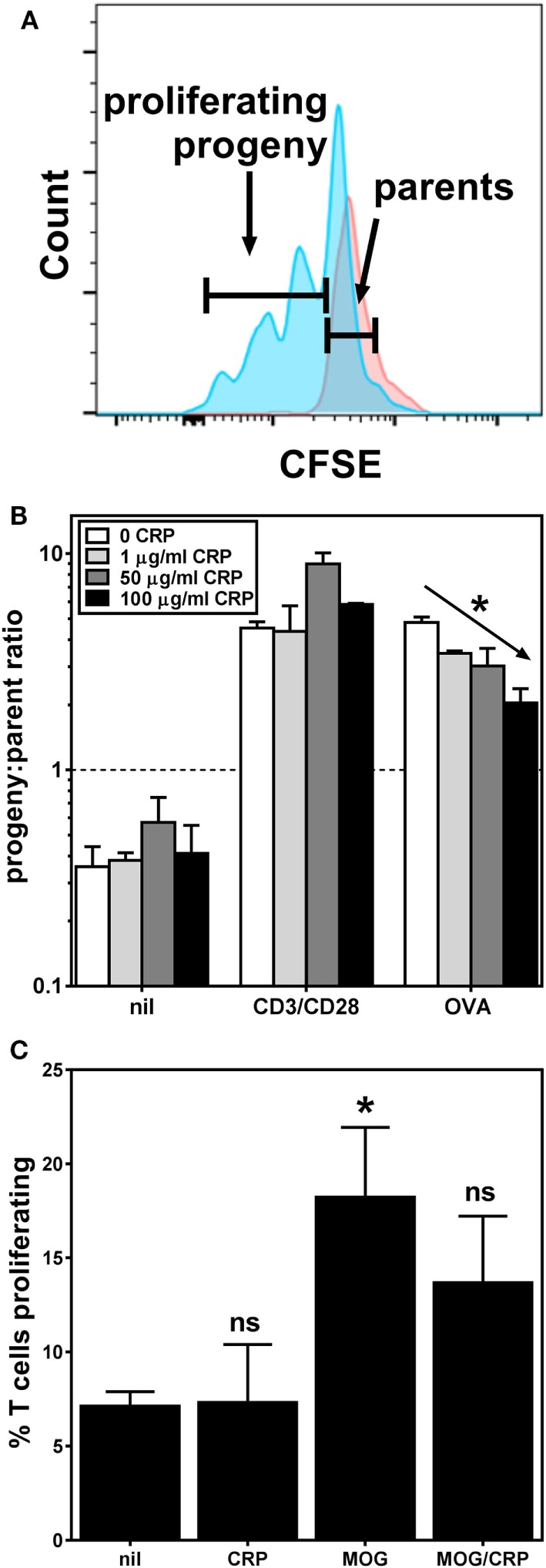
C-reactive protein (CRP) inhibits bone marrow-derived dendritic cell (BMDC)-mediated/antigen-driven T cell proliferation. **(A)** Typical flow cytometry histograms for carboxyfluorescein succinimidyl ester-labeled OT-II T cells harvested 3 days after co-culture with BMDCs without antigen (parental generation, red) and with BMDCs loaded with OVA_323–339_ peptide (progeny generations, blue). **(B)** Proliferation of OT-II T cells co-cultured with antigen-naïve BMDCs and no other stimulant (nil), or with anti-CD3ε/anti-CD28 antibodies (CD3/CD28), and co-cultured with OVA_323–339_ peptide-loaded BMDCs (OVA), without or with addition of CRP. The diagonal arrow indicates *p* < 0.0001 (*) for a linear trend test of column means in left-to-right column order. **(C)** 2D2 T cell proliferation in the presence of antigen-naïve BMDCs without (nil) or with 50 µg/ml CRP, or in the presence of myelin oligodendrocyte glycoprotein (MOG)_35–55_ peptide-loaded BMDCs without (MOG) or with 50 µg/ml CRP (MOG/CRP). The symbols indicate not significant or *p* < 0.05 (*) for one-way analyses of variance with Tukey’s multiple comparisons tests compared to nil (*n* = 3–6 per group).

We next sought to determine if the observed effects of CRP on BMDC activation/maturation phenotype and cytokine production affects their T cell stimulatory function. We found that CRP (1–100 µg/ml) had no significant effect on the proliferation of OT-II T cells co-cultured with BMDCs in the absence of any stimulus (Figure 3B; nil) or in the presence of T cell activating antibodies (Figure 3B; CD3/CD28). Importantly, however, when BMDCs loaded with OVA_323–339_ peptide were used as APCs, the addition of CRP caused a dose-dependent inhibition of OT-II T cell proliferation (Figure 3B; OVA). Using the MOG TCR-transgenic model (2D2) we obtained similar results, i.e., CRP (50 µg/ml) significantly inhibited the proliferation of 2D2 T cells co-cultured with BMDCs loaded with MOG_35–55_ peptide (Figure 3C). These data confirm that CRP’s prohibition of BMDC activation/maturation and cytokine production reduces their ability to stimulate antigen-specific T cell proliferation. The fact that in both model systems, CRP had no effect on T cells directly stimulated with anti-CD3ε/anti-CD28 antibodies shows that CRP’s influence on T cell proliferation must be *via* its actions on BMDCs.

### CRP Does Not Prohibit the Activation/Maturation of FcγRIIB^−/−^ BMDCs

**Figure 4:**
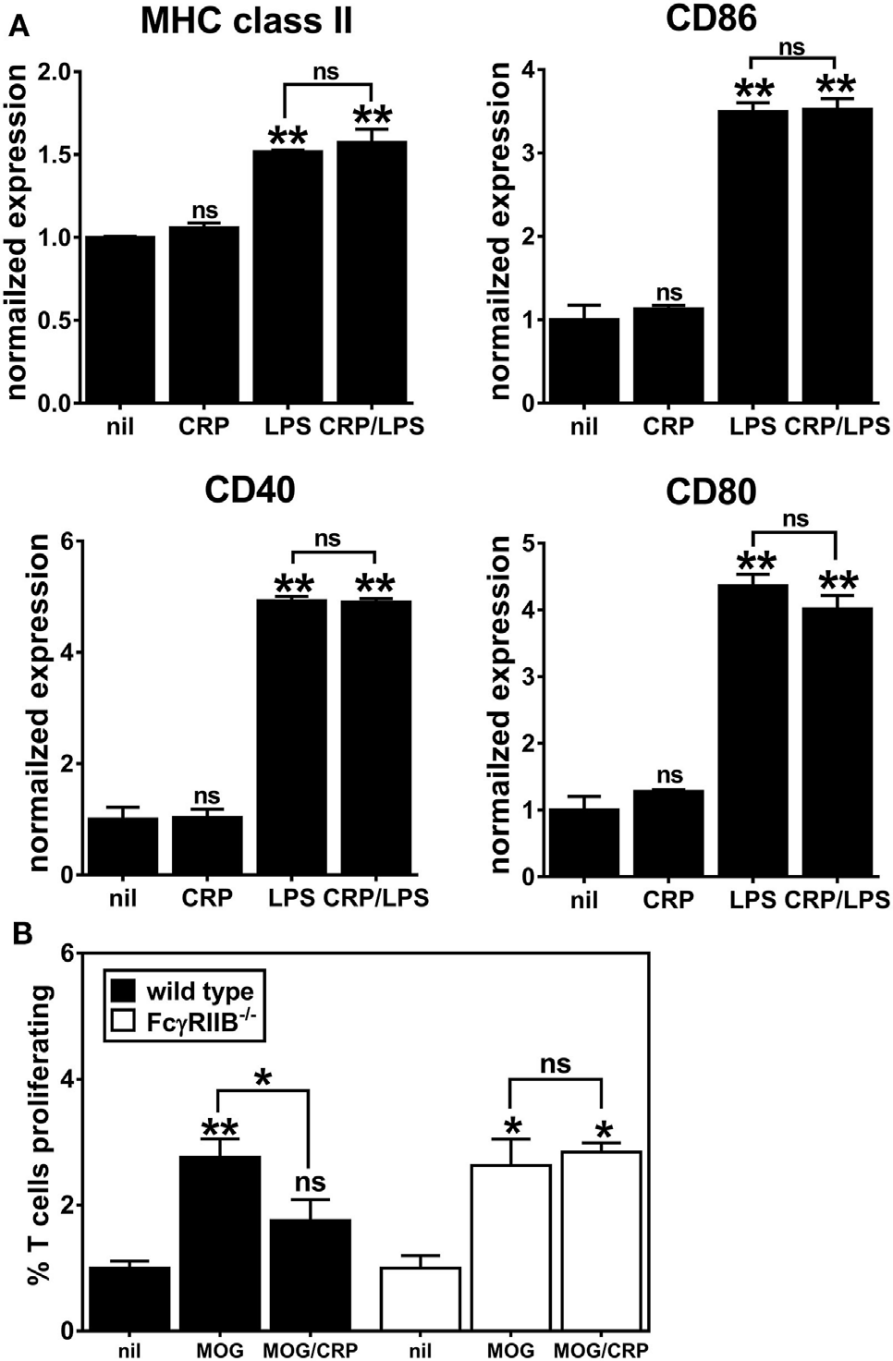
C-reactive protein (CRP)-mediated prohibition of lipopolysaccharide (LPS)-induced maturation of bone marrow-derived dendritic cells (BMDCs) and inhibition of BMDC-mediated/antigen-driven proliferation of 2D2 T cells is FcγRIIB-dependent. **(A)** Surface expression of MHC class II, CD86, CD40, and CD80 on FcγRIIB^−/−^ BMDCs left untreated (nil), or treated with CRP (50 µg/ml), LPS (1 µg/ml), or CRP and LPS. Each marker’s expression is normalized to the nil group and the symbols directly above each bar indicate not significant (ns), *p* < 0.05 (*), or *p* < 0.005 (**) compared to nil. There was no significant difference between the LPS versus CRP/LPS groups. One-way analyses of variance (ANOVA) with Tukey’s multiple comparisons tests (*n* = 3 experiments). **(B)** 2D2 T cell proliferation in the presence of wild type versus FcγRIIB^−/−^ BMDCs. BMDCs were antigen-naïve (nil) or myelin oligodendrocyte glycoprotein (MOG)_35–55_ peptide-loaded and CRP was at 50 µg/ml. The symbols above the bars indicate ns, *p* < 0.05 (*) and *p* < 0.005 (**). The symbols above the brackets compare the MOG/CRP versus MOG groups. One-way ANOVA with Tukey’s multiple comparisons tests (*n* = 3 per group).

C-reactive protein binds to both activating and inhibitory Fc receptors, thereby triggering a diversity of cellular responses *in vitro* (2, 21) and many of the *in vivo* biological actions of human CRP in CRPtg are fully supported by FcγRIIB (6, 22). Since FcγRs *per se*, and FcγRIIB in particular, are widely expressed by both human and mouse DCs (7), we generated DCs using FcγRIIB^−/−^ BM to test if CRP’s influence on BMDC phenotype and function required FcγRIIB. Like the expression on immature WT BMDCs (Figure 2A), expression of MHC class II, CD80, CD40, and CD86 on immature FcγRIIB^−/−^ BMDCs was unaffected by CRP alone (50 µg/ml), and LPS triggered their increase (Figure 4A). However, in stark contrast to its effect on LPS-matured WT BMDCs (Figure 2A), CRP did not impair the LPS-triggered upregulation of MHC class II, CD86, and CD40 by FcγRIIB^−/−^ BMDCs (Figure 4A). Like for WT BMDCs, expression of IL-10 by LPS-treated FcγRIIB^−/−^ BMDCs (308.5 ± 12.5 ng/ml) was lowered by CRP (69.6 ± 8.7 ng/ml). However, unlike for WT BMDCs, for FcγRIIB^−/−^ BMDCs treated with LPS the amount of IL-12p70 produced (948.9 ± 25.3 ng/ml) was not reduced by CRP (1017.1 ± 51.6 ng/ml). These findings strongly suggest that FcγRIIB expression is required for CRP to prohibit LPS-induced activation/maturation of BMDCs and to suppress production of the T cell stimulatory cytokine IL-12p70. As expected, when MOG_33–55_ peptide-loaded FcγRIIB^−/−^ BMDCs were used as APCs, CRP (50 µg/ml) did not impair their proliferation (Figure 4B). In our hands, FcγRIIB^−/−^ BMDCs did not stimulate OT-II T cell proliferation even when loaded with OVA_323–339_ (data not shown), precluding us from assessing if CRP requires FcγRIIB in the OT-II model system. Nevertheless, the results from the 2D2 model confirmed that CRP’s ability to prohibit BMDC stimulation of an antigen-specific T cell response is facilitated by FcγRIIB expressed on BMDCs.

### Transgenic Expression of Human FcγRIIB Supports Human CRPs Actions on Mouse FcγRIIB^−/−^ BMDCs

**Figure 5:**
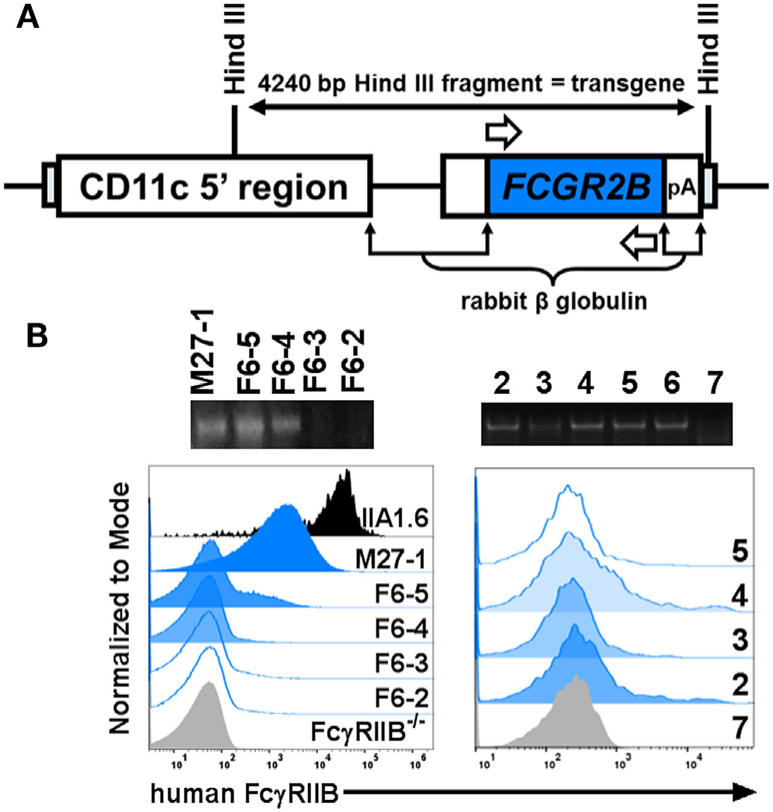
Generation of _cd11c_FcγRIIB^hu^ mice. **(A)** The targeting vector (fully described in (8)) encodes the mouse CD11c minimal promoter driving the human *FCGR2B* open reading frame. **(B)** Left panels: agarose gel electrophoresis of PCR amplified gDNA from a male (M27-1) and four female (F6-2, -3, -4, and -5) potential founders; three of which carried the *FCGR2B* transgene as indicated by presence of a 658 base pair amplicon generated using human *FCGR2B*-specific primers [white arrows in panel **(A)**]. Expression of human FcγRIIB was detected on mouse peripheral blood cells on M27-1, F6-5, and F6-5, using flow cytometry with an anti-human FcγRIIB antibody (clone AT 10). Murine B cell lymphoma (IIA1.6) cells transfected with a plasmid containing cDNA encoding human FcγRIIB (fully described in (24)) and peripheral blood cells from an FcγRIIB^−/−^ mouse served as controls. Right panels: agarose gel electrophoresis of PCR amplified gDNA from six littermates descended from F6-5 (which transmitted the transgene). Their respective expression of human FcγRIIB on CD11c^+^ splenocytes is uniform.

The apparent requirement of mouse FcγRIIB for human CRP-mediated prohibition of BMDC activation/maturation and 2D2 T cell proliferation prompted us to investigate this biology further. Accordingly, we generated FcγRIIB^−/−^ mice that express a human *FCGR2B* transgene. Expression of the human FcγRIIB receptor was restricted to DCs by using a vector that contains the CD11c minimal promoter (kindly provided by Dr. Thomas Brocker, Institute for Immunology, LMU Munich Goethestr. 31, D-80336 Munich, Germany) (8). Briefly, a full-length cDNA clone encoding human *FCGR2B* (23) was inserted into the vector (Figure 5A) to drive *FCGR2B* expression on CD11c^+^ DCs in all mouse tissues. Transgenic mice (_cd11c_FcγRIIB^hu^) were then established by injecting the construct directly into fertilized FcγRIIB^−/−^ eggs in the UAB Transgenic & Genetically Engineered Models Core. Offspring were screened for presence of the human transgene by PCR and flow cytometry was used to detect surface expression of human FcγRIIB on peripheral blood mononuclear cells (Figure 5B, left). Of the three potential founders identified (M27-1, F6-5, and F6-4; Figure 5B, left), only one (F6-5) showed germline transmission of the transgene. Transgenic descendants of F6-5 showed uniform expression of human FcγRIIB (Figure 5B, right) and were used for all further experiments.

**Figure 6:**
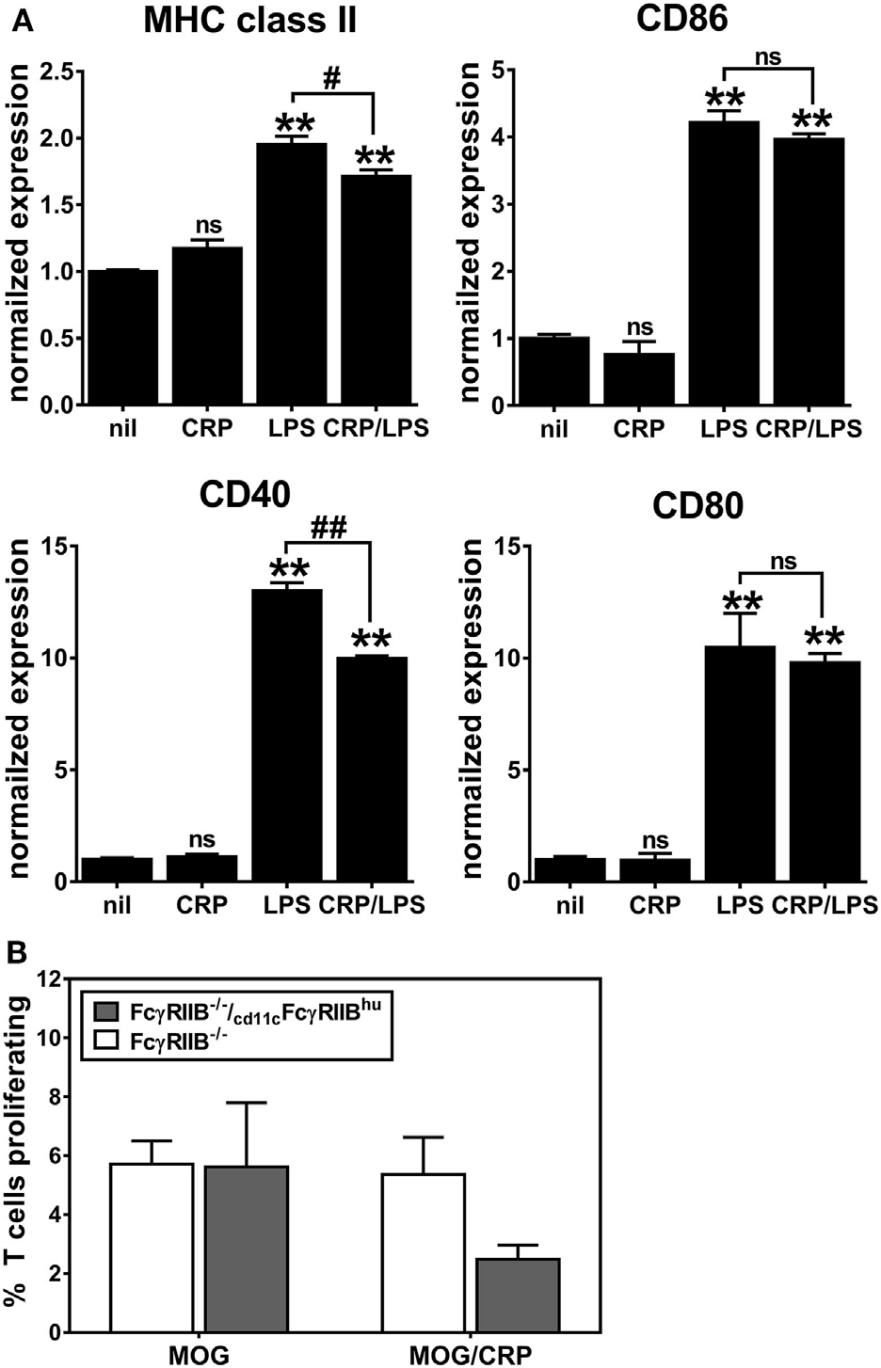
CD11c-specific expression of human FcγRIIB reconstitutes C-reactive protein (CRP)-mediated suppression of **(A)** expression of MHC class II and CD40 on lipopolysaccharide (LPS)-matured bone marrow-derived dendritic cells (BMDCs) and **(B)** BMDC-mediated/myelin oligodendrocyte glycoprotein-driven 2D2 T cell proliferation. The symbols directly above each bar indicate not significant or *p* < 0.005 (**) compared to nil. The symbols above each bracket indicate *p* < 0.05 (#), or *p* < 0.005 (##) comparing LPS versus CRP/LPS groups. One-way analyses of variance with Tukey’s multiple comparisons tests (*n* = 3–6 per group).

We generated _cd11c_FcγRIIB^hu^ BMDCs and confirmed that they upregulated expression of MHC class II, CD86, CD40, and CD80 after LPS-triggered activation/maturation (Figure 6A) and that CRP alone had no effect on expression of these markers (Figure 6A). Expression of human FcγRIIB partly reconstituted the CRP prohibitory effect on BMDC maturation, i.e., upon LPS-stimulation, CRP prohibited the expression of MHC class II and CD40 (Figure 6A). CRP inhibited IL-10 production by LPS-stimulated _cd11c_FcγRIIB^hu^ BMDCs (459.4 ± 3.1 ng/ml without CRP and no detectable amounts with CRP), but not IL-12p70 production (485.9 ± 94.8 ng/ml and 689.9 ± 235.9 ng/ml without or with CRP, respectively). Although the effect was not significant (ns), when MOG_35–55_ peptide-loaded _cd11c_FcγRIIB^hu^ BMDCs were used as APCs, their ability to stimulate the proliferation of 2D2 T cells was reduced by CRP (Figure 6B). These data generally support the premise that CRP’s influence on DCs requires their expression of FcγRIIB, since some of the effects of CRP on FcγRIIB^−/−^ BMDCs are recovered by expression of human FcγRIIB. Interestingly, although reconstitution of FcγRIIB^−/−^ BMDCs with human FcγRIIB restored their ability to promote OVA_323–339_-driven OT-II T cell proliferation, CRP (50 µg/ml) did not have a significant effect (data not shown).

### Human FcγRIIB Supports Human CRP’s Protective Actions in EAE

**Figure 7:**
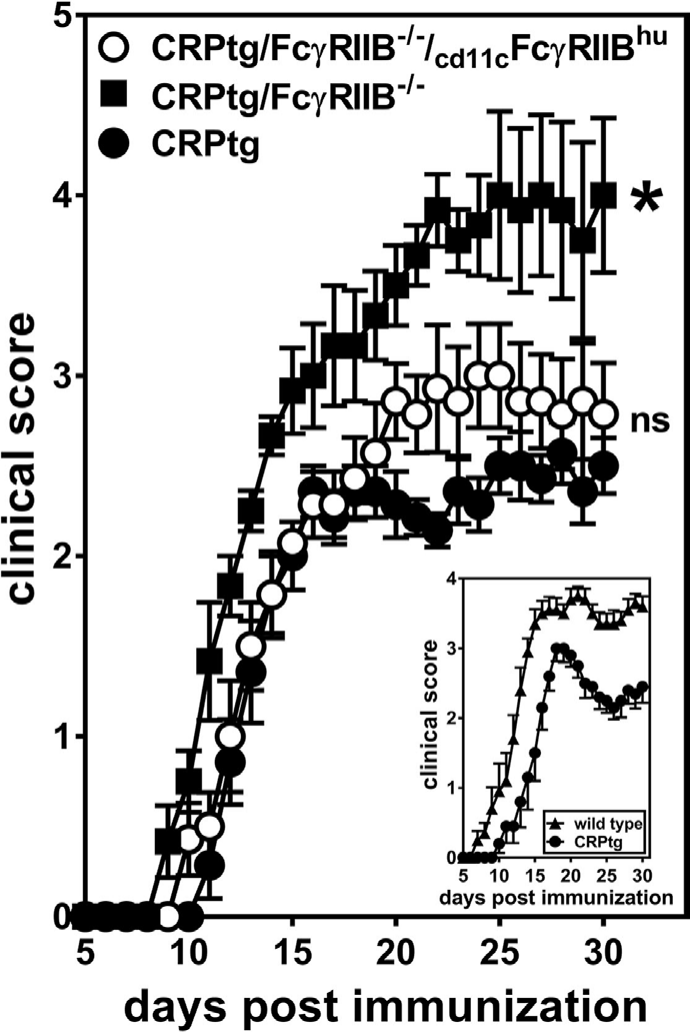
CD11c-specific expression of human FcγRIIB restores resistance to experimental autoimmune encephalomyelitis (EAE) in CRP transgenic mice (CRP)tg/FcγRIIB^−/−^ mice. Mice were immunized with myelin oligodendrocyte glycoprotein_35–55_ and their ensuing EAE symptoms were monitored for 30 days for CRPtg (●), CRPtg/FcγRIIB^−/−^ (■), and CRPtg/FcγRIIB^−/−^/_cd11c_FcγRIIB^hu^ (**⚪**). The asterisk indicates the course of disease in CRPtg/FcγRIIB^−/−^ is significantly worse (see Table 1 for details). The inset shows the course of EAE in CRPtg compared to wild type from a separate experiment (*n* = 6–10 mice per group).

We had previously shown that CRPtg undergoing EAE have delayed onset and reduced severity of disease compared to WT and that this beneficial effect of CRP is FcγRIIB-dependent (5, 6, 20, 25), and herein we provide new evidence that this FcγRIIB-dependency extends to BMDCs *in vitro*. Moreover, although not all the observed effects of human CRP on BMDCs were supported by human FcγRIIB, CD11c-specific expression of human FcγRIIB was sufficient to fully reconstitute human CRP’s beneficial actions in EAE (Figure 7; Table 1). Given that human CRP can utilize human FcγRIIB expressed by CD11c^+^ cells in transgenic mice, it is possible that the same or a similar CRP→FcγRIIB pathway operates in humans to regulate tolerance and prevent autoimmunity.

**Table 1:** Experimental autoimmune encephalomyelitis (EAE) outcomes in C-reactive protein transgenic mice (CRPtg) lacking mouse FcγRIIB and/or expressing human FcγRIIB.

Genotype (*n*)	Day of onset^a^ (mean ± SEM)	CDI^b^ (mean ± SEM)	Maximum score^c^ (mean ± SEM)
CRPtg (7)	14.0 ± 0.43	41.71 ± 0.94	2.86 ± 0.09
CRPtg/FcγRIIB^−/−^ (6)	11.67 ± 0.33^d^	67.17 ± 5.15^d^	4.17 ± 0.40^d^
CRPtg/FcγRIIB^−/−^/_cd11c_FcγRIIB^hu^ (7)	13.71 ± 0.52 ^ns^	48.43 ± 3.79 ^ns^	3.43 ± 0.28^ns^
Analysis of variance	*p* = 0.004	*p* = 0.0005	*p* = 0.0135

*^a^The day the clinical score attained a value ≥2 and remained ≥2 for at least 2 days*.

*^b^Cumulative disease index: the sum of clinical scores from day 0–31*.

*^c^The maximum clinical score attained by each mouse. Mice that succumbed to EAE were assigned a score of 6*.

*^d^Tukey’s multiple comparisons test, p < 0.05*.

*^ns^Tukey’s multiple comparisons test, not significant (*p* > 0.05)*.

## Discussion

Previously, we showed that human CRP protects CRPtg mice from EAE triggered either directly by immunization with MOG or indirectly by the transfer of MOG-specific T cells (5, 20, 25) and that this protection was FcγRIIB-dependent manner (6). Although human CRP can have direct effects on T cells (25), the initial evidence of CRP inhibiting EAE suggested that CRP most likely conferred protection by acting on an intermediary APC. This study provides strong evidence to this effect, i.e., *in vivo* human CRP protects mice from EAE by acting on CD11c^+^ FcγRIIB-expressing DCs. We propose that the beneficial effect of transgenically expressed human CRP in EAE, and perhaps other T cell-mediated diseases like lupus and collagen-induced arthritis (26–37), is achieved *via* its capacity to inhibit DC development and function, thereby diminishing the stimulation of pathogenic T cells.

Our *in vitro* data reveal several separate, but likely additive, mechanisms by which CRP impacts the T cell stimulating actions of DCs. First, CRP dose-dependently decreased the propor-tion of BM progenitors that developed into BMDCs, suggesting that CRP influences the fate of hematopoietic stem cells. Native pentameric CRP is likely required for this effect as heat denatured CRP did not have any effect (data not shown). Furthermore, CRP did not significantly affect early or late apoptosis or necrosis during the course of BM culture, demonstrating that CRP binding to phosphatidylserine on dying cells does not play a significant role and that CRP’s influence is likely not due to selective killing of certain BM progenitors. Indeed, in separate studies we have also observed that CRP dose-dependently promotes the development of myeloid-derived suppressor cells (MDSC) at the expense of DCs (Figure S1 in Supplementary Material), and that the spleens of healthy CRP knockout mice (27) have an increased number of plasmacytoid (CD11c^+^ CD11b^+/−^ Siglec H^+^) and conventional (CD11c^+^ CD11b^+^ Siglec H^−^) DCs compared to WT and CRPtg (Figure S2 in Supplementary Material). The mechanism by which CRP alters myeloid progenitor cell developmental fate is still under investigation, but the fact that CRP shifts the myeloid balance away from DCs (which can promote T cell proliferation) and toward MDSCs (which can suppress it) directly implicates CRP in the regulation of the balance between adaptive immunity and tolerance. Second, CRP dose-dependently prohibits the LPS-triggered (TLR4-triggered) activation/maturation of BMDCs as evidenced by its ability to limit expression of MHC class II and co-stimulatory markers. Notably, CRP had no effect in the absence of a maturation signal (i.e., immature BMDCs) or in the presence of the TLR9 agonist CpG oligodeoxynucleotides (data not shown). This implies that *in vivo* CRP attenuates the responses of mature DCs in the periphery (i.e., those not participating in central tolerance) and does not impact immature DCs. Third, CRP impairs the production of IL-10 and IL-12p70 by BMDCs, two pleiotropic cytokines that can suppress (29) or promote (30) T cell functions, respectively. Fourth, CRP inhibited the ability of peptide-loaded mature BMDCs to stimulate antigen-driven T cell proliferation. Unexplored was whether CRP impairs the ability of BMDCs to uptake, process, and present antigen, but others have shown that CRP can also impact these processes (31–33).

We previously showed that in the absence of FcγRIIB, human CRP cannot protect mice against EAE (6). That observation led us here to test whether the CRP-responsive, FcγRIIB-expressing cell that might promote CRP’s beneficial effects in EAE are DCs (7). In preliminary studies, we showed that CRP dose-dependently decreased the yield of both WT and FcRγ^−/−^ CD11c^+^ BMDCs, but not FcγRIIB^−/−^ ones (Figure S3 in Supplementary Material). In alignment with those initial data, we showed herein that FcγRIIB^−/−^ BMDCs maintain their ability to mature in response to LPS and to subsequently stimulate 2D2 T cell proliferation when loaded with MOG_35–55_, but are refractory to inhibition by CRP. Importantly, in the absence of FcγRIIB expression, CRP was unable to downregulate BMDC production of the T cell stimulating cytokine IL-12p70. These findings highlight the importance of FcγRIIB for the inhibitory action of CRP on the development, maturation, cytokine production, and antigen-specific T cell stimulatory function of BMDCs. Since, human CRP can bind both mouse and human FcγRs *in vitro* and *in vivo* (2, 21), we generated a mouse completely deficient in endogenous mouse FcγRIIB, but expressing human *FCGR2B* on CD11c^+^ cells. Using bone marrow from these _cd11c_FcγRIIB^hu^ mice we showed that human CRP utilized human FcγRIIB to evoke impairment of BMDC activation and T cell stimulating function, but not to regulate IL-12p70 production. Nevertheless, CRP protection from EAE was fully reconstituted in CRPtg/_cd11c_FcγRIIB^hu^ mice. We recognize that mouse CD11c, and, therefore, human FcγRIIB in the _cd11c_FcγRIIB^hu^ mice, might be expressed at low levels on cell types other than DCs and that other DC subtypes may not express CD11c at all [e.g., plasmacytoid DCs and DCs with tolerogenic phenotypes (34)]. Nevertheless, this study is the first to show that human CRP interaction with human FcγRIIB expressed *in vivo* on CD11c^+^ cells can modulate EAE.

We suspect that CRP regulates the generation and actions of DCs in the periphery (i.e., those not directly involved in central tolerance), thereby limiting the activation of auto-reactive T cells especially in the setting of tolerance breakdown. Withal, CRP promotes the number and generation of myeloid-derived suppressor cells (MDSCs) [Figure S1 in Supplementary Material (35)], a cell type known to potently suppress T cell proliferation (36). Simply by modulating the myeloid lineage development away from DCs and toward MDSCs, CRP could thus profoundly impact T cell immunity and the maintenance of peripheral tolerance. This role is unlikely to be restricted to EAE/MS and should also be manifest in the setting of immunosenescence and aging, for example [reviewed in Ref. (37)]. Indeed, some of the prominent features of immunosenescence are inflammation, decreased T cell numbers, and decreased naïve and memory T cell responsiveness (37, 38, 39), and in the aged, inflammaging can contribute to dysregulated DC responses and a consequent breakdown of tolerance that can predispose them to autoimmunity (40, 41). We propose that in this context, modest elevation of CRP due to biological aging (12) might act as a tonic suppressor of DC activation and thus limit auto-reactivity.

## Ethics Statement

This study was carried out in accordance with the recommendations of the *Guide for the Care and Use of Laboratory Animals; Eighth Edition* (NIH Academies Press, 2011) and the Institutional Animal Care and Use Committees at the University of Alabama at Birmingham.

## Author Contributions

AS, RJ, TW, and NJ designed the experiments and RJ, TW, and NJ performed them. JW and AG aided in the generation of the _cd11c_FcgRIIB^hu^ mouse. RJ and AS wrote the manuscript.

## Conflict of Interest Statement

The authors declare that the research was conducted in the absence of any commercial or financial relationships that could be construed as potential conflict of interest.
