# Supplementary material for: C-Reactive Protein Impairs Dendritic Cell Development, Maturation, and Function: Implications for Peripheral Tolerance
Source: Front Immunol. 2018 Mar 5;9:372. doi: 10.3389/fimmu.2018.00372 (PMC5845098; doi:10.3389/fimmu.2018.00372)
Supplement: Supplementary file 1 [file data_sheet_1.docx]

Supplementary Material:

C-reactive protein impairs dendritic cell activation: implications for peripheral tolerance.

Rachel V. Jimenez, Tyler T Wright, Nicholas R. Jones, Jianming Wu, Andrew W. Gibson, and Alexander J. Szalai*

***Correspondence**: Alexander J. Szalai: [aszalai@uabmc.edu](mailto:aszalai@uabmc.edu)

## Supplementary Figures


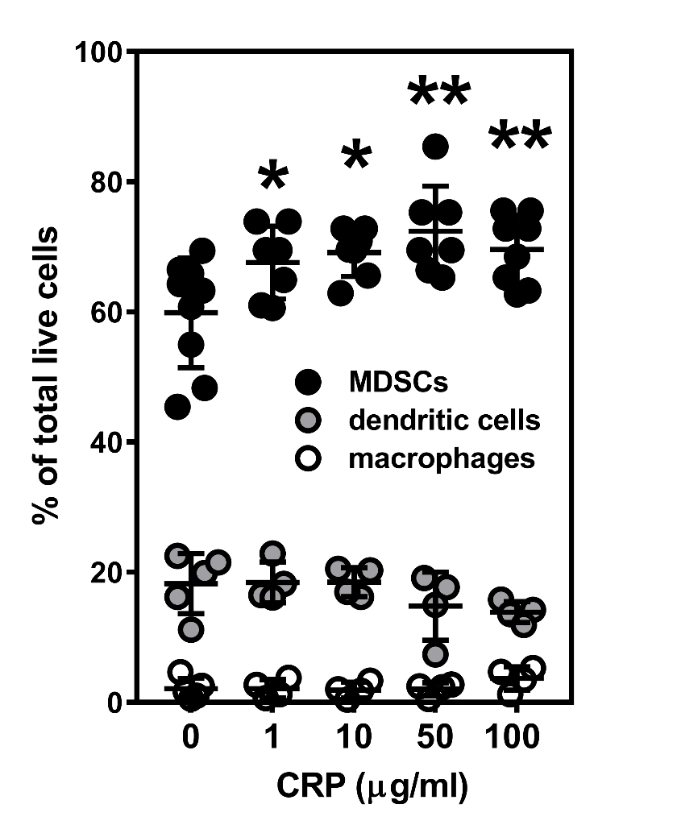


Figure S1. Human CRP promotes the generation of bone marrow-myeloid derived suppressor cells (BM-MDSC). Bone marrow was cultured under conditions favoring MDSC generation (1) with human CRP added on day 0. CRP significantly and dose-dependently increased the proportion of MDSCs (●, CD11c^-^ CD11b^+^ F4/80^-^ Ly6C^+^ Ly6G^+^), at the expense of dendritic cells (●, CD11c^+^ CD11b^+^ F4/80^-^), with no effect on macrophages (○, CD11c^-^ CD11b^+^ F4/80^-^). One-way *ANOVA* with multiple comparisons *p* < 0.01 (*) and *p* < 0.001 (**) from n = 7 – 10 cultures.


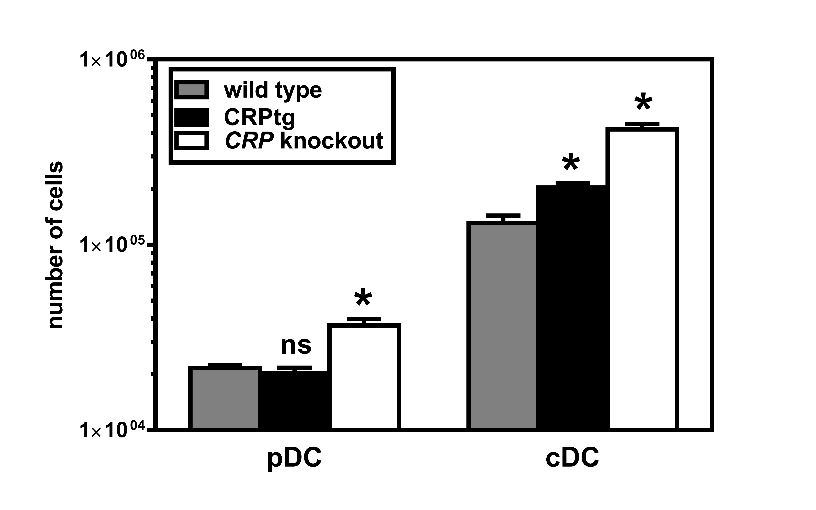


**Figure S2.** Loss of CRP expression *in vivo* alters DC subtypes. In the absence of CRP (*i.e.* in *CRP* knockout mice (2)), the numbers of both plasmacytoid DCs (CD11c^+^ CD11b^+/-^ Siglec H^+^) and conventional DCs (CD11c^+^ CD11b^+^ Siglec H^-^) are increased in the spleen. The latter play a major role in CD4^+^ T cell activation. One-way *ANOVA* with multiple comparisons *p* < 0.05 (*) from n = 3 mice per genotype.


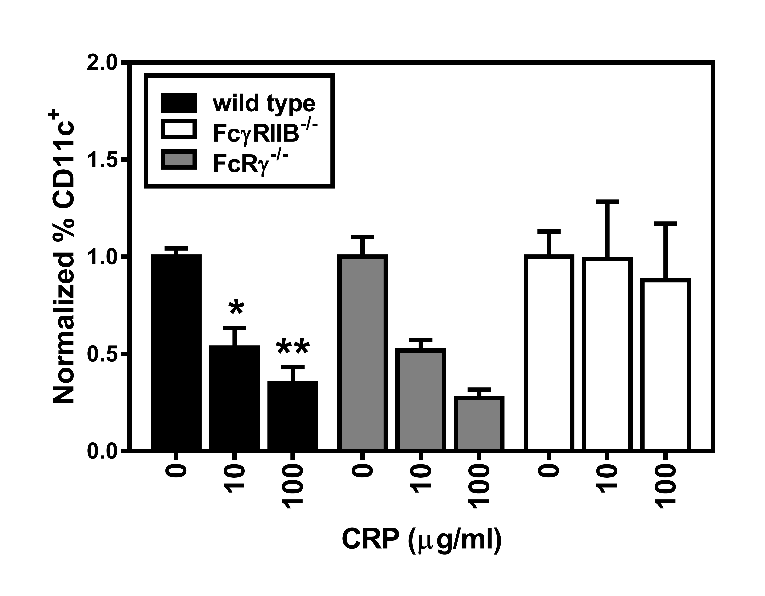


**Figure S3.** CRP impairs the generation of CD11c^+^ BMDCs in an FcγRIIB-dependent manner. BMDCs were generated with bone marrow from wild type, FcγRIIB^-/-^, and FcRγ^-/-^ mice. The latter lack the common gamma chain used for signaling by activating FcγRs (FcγRI, FcγRIII, and FcγRIV) (3). CRP significcantly and dose-dependently decreased the proportion of CD11c^+^ BMDCs generated using wild type bone marrow with a similar trend (not significant) for FcRγ^-/-^; this CRP effect is absent in FcγRIIB^-/-^ cultures. Each genotype is normalized to cultures that were never exposed to CRP. Two-way ANOVA with Dunnett’s multiple comparisons test versus 0 CRP within each genotype p < 0.05 (*) and p < 0.005 (**) from n = 3 – 9 experiments.

1. Hochst B, Mikulec J, Baccega T, Metzger C, Welz M, Peusquens J, et al. Differential induction of Ly6G and Ly6C positive myeloid derived suppressor cells in chronic kidney and liver inflammation and fibrosis. *PloS one* (2015) 10(3):e0119662. doi: 10.1371/journal.pone.0119662. PubMed PMID: 25738302; PubMed Central PMCID: PMC4349817.

2. Jones NR, Pegues MA, McCrory MA, Kerr SW, Jiang H, Sellati R, et al. Collagen-induced arthritis is exacerbated in C-reactive protein-deficient mice. *Arthritis and rheumatism* (2011) 63(9):2641-50. doi: 10.1002/art.30444. PubMed PMID: 21567377; PubMed Central PMCID: PMC3168703.

3. Takai T, Li M, Sylvestre D, Clynes R, Ravetch JV. FcR gamma chain deletion results in pleiotrophic effector cell defects. *Cell* (1994) 76(3):519-29. Epub 1994/02/11. PubMed PMID: 8313472.
